# Utilization of a Directly Supervised Telehealth-Based Exercise Training Program in Patients With Nonalcoholic Steatohepatitis: Feasibility Study

**DOI:** 10.2196/30239

**Published:** 2021-08-17

**Authors:** Victoria Motz, Alison Faust, Jessica Dahmus, Benjamin Stern, Christopher Soriano, Jonathan G Stine

**Affiliations:** 1 Penn State Milton S Hershey Medical Center Hershey, PA United States

**Keywords:** physical activity, fatty liver, telemedicine, liver, nonalcoholic fatty liver disease, liver disease, fatty liver disease, aerobic training, telehealth, fitness, feasibility, steatohepatitis

## Abstract

**Background:**

Most patients with nonalcoholic fatty liver disease (NAFLD) are physically inactive despite the well-known benefits of physical activity. Telehealth offers promise as a novel way to deliver an exercise training program and increase physical activity. However, the feasibility, safety, and efficacy of telehealth-based exercise programs is unknown in patients with NAFLD.

**Objective:**

The aim of this study was to determine the feasibility of a directly supervised exercise training program delivered exclusively with telehealth to patients with nonalcoholic steatohepatitis (NASH), the progressive form of NAFLD.

**Methods:**

In response to COVID-19 research restrictions, we adapted an existing clinical trial and delivered 20 weeks of moderate-intensity aerobic training 5 days a week under real-time direct supervision using an audio–visual telehealth platform. Aerobic training was completed by walking outdoors or using a home treadmill. Fitness activity trackers with heart rate monitors ensured exercise was completed at the prescribed intensity with real-time feedback from an exercise physiologist.

**Results:**

Three female patients with biopsy-proven NASH were enrolled with a mean age of 52 (SD 14) years. The mean body mass index was 31.9 (SD 5.1) kg/m2. All patients had metabolic syndrome. All patients completed over 80% of exercise sessions (mean 84% [SD 3%]) and no adverse events occurred. Body weight (mean –5.1% [SD 3.7%]), body fat (mean –4.4% [SD 2.3%]), and waist circumference (mean –1.3 in. [SD 1.6 in.]) all improved with exercise. The mean relative reduction in magnetic resonance imaging-proton density fat fraction (MRI-PDFF) was 35.1% (SD 8.8%). Mean reductions in hemoglobin A1c and Homeostatic Model Assessment for Insulin Resistance were also observed (–0.5% [SD 0.2%] and –4.0 [SD 1.2], respectively). The mean peak oxygen consumption (VO2peak) improved by 9.9 (SD 6.6) mL/kg/min.

**Conclusions:**

This proof-of-concept study found that supervised exercise training delivered via telehealth is feasible and safe in patients with NASH. Telehealth-based exercise training also appears to be highly efficacious in patients with NASH, but this will need to be confirmed by future large-scale trials.

**Trial Registration:**

ClinicalTrials.gov NCT03518294; https://clinicaltrials.gov/ct2/show/NCT03518294

## Introduction

To date, there is no effective drug therapy nor cure for nonalcoholic fatty liver disease (NAFLD) or its progressive form, nonalcoholic steatohepatitis (NASH). Lifestyle modification, which includes both dietary change and increasing physical activity, remains the most effective treatment for NAFLD and is recommended for all patients. Despite physical activity’s well-known benefits, over 80% of patients with NAFLD are physically inactive [1,2]. Consequently, disease progression is common. There is a clear unmet need to increase physical activity in order to improve patient outcomes, especially in light of the rapidity of weight gain from increased sedentary behavior attributable to the novel COVID-19 pandemic [3]. Out of necessity, telehealth has emerged at the forefront of health care delivery during the pandemic. Telehealth offers additional promise for patients with NAFLD to (1) remove self-identified barriers preventing physical activity [1] and (2) deliver an exercise training program. However, telehealth-delivered exercise training programs remain unexplored in patients with NAFLD and their feasibility, safety, and efficacy are unknown.

## Methods

Because institutional restrictions prevented in-person clinical trial activity at the height of the pandemic, we adapted an existing clinical trial that was actively recruiting patients (prior to COVID-19 restrictions, 25 patients were recruited under the original clinical trial protocol) [4] and delivered 20 weeks of moderate-intensity aerobic training 5 days a week under real-time direct supervision by an exercise physiologist using an Institutional Review Board–approved audio–visual (A–V) telehealth platform with 2-way communication. Aerobic training was completed by walking outdoors or using a home treadmill. Fitness activity trackers with heart rate monitors ensured exercise was completed at the prescribed intensity with real-time feedback from an exercise physiologist. Each exercise session lasted 30 minutes, and was preceded by a warm-up and ended with a cool-down in accordance with the original study protocol. Feasibility was defined as completing 80% or more of exercise sessions [5]. Secondary clinical outcomes were captured according to the existing study protocol [4]. Patients also received dietary counseling according to the original study protocol which allowed for telehealth as a way to provide the nutritional feedback.

## Results

**Figure 1:**
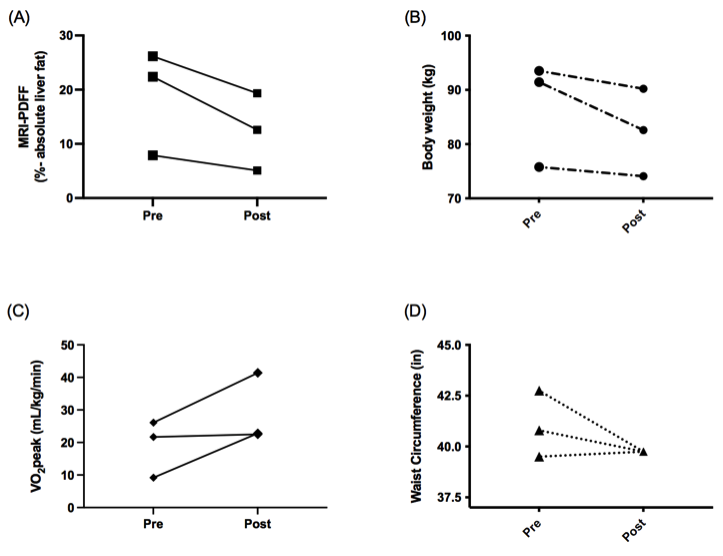
Observed clinical benefits of a supervised 20-week telehealth-delivered exercise training program. (A) All patients reduced MRI-PDFF-measured liver fat with exercise training. (B) Exercise training reduced body weight in all patients. (C) Cardiorespiratory fitness improved in all patients (mean VO2 peak +9.9 [SD 6.6] mL/kg/min). (D) Waist circumference was reduced by a mean 1.3 (SD 1.6) in. MRI-PDFF: magnetic resonance imaging proton density fat fraction; VO2 peak: peak oxygen consumption.

Three patients with biopsy-proven NASH were recruited and enrolled during the period of COVID-19 research restrictions. All patients were female with a mean age of 52 (SD 14) years. The mean body mass index was 31.9 (SD 5.1) kg/m^2^. All patients had metabolic syndrome. Liver histology was as follows: NAFLD activity score of 4 (n=2) and 5 (n=1); and fibrosis stage of 1 (n=2) and 0 (n=1). All patients completed 80% or more of exercise sessions (mean 84% [SD 3%]) and no adverse events occurred. Body weight (mean –5.1% [SD 3.7%]), body fat (mean –4.4% [SD 2.3%]), and waist circumference (mean –1.3 [SD 1.6] in.) all improved with exercise (Figure 1). The mean relative reduction in liver fat measured by magnetic resonance imaging-proton density fat fraction (MRI-PDFF) was 35.1% (SD 8.8%). Mean reductions in hemoglobin A1c, aspartate aminotransferase, alanine aminotransferase, Homeostatic Model Assessment for Insulin Resistance were –0.5% (SD 0.2%), –8.5 (SD 3.2) IU/L, –12.5 (SD 6.7) IU/L, and –4.0 (SD 1.2), respectively. The mean peak oxygen consumption (VO_2_peak) improved by 9.9 (SD 6.6) mL/kg/min. Owing to the small sample size, no change in secondary clinical outcomes achieved statistical significance (*P*>.05).

## Discussion

This proof-of-concept study found supervised exercise training delivered via an A–V telehealth platform to be feasible and safe in patients with NASH. All patients met the a priori definition of feasibility and none experienced an adverse event. Importantly, remote exercise training also appears to be efficacious in patients with NASH. The observed reduction in MRI-PDFF–measured liver fat, insulin resistance, body weight, and body fat in parallel with gains in physical fitness even exceed those that are published for supervised in-person exercise training programs of similar length [2]. While we look to future large-scale studies to validate the efficacy of this small study, these data are nonetheless promising and further suggest that A–V telehealth has a role in the routine care of patients with NASH.

While this is the first study to employ remote supervision using real-time A–V telehealth technology to deliver an exercise training program in patients with NASH, other studies have used web-based or mobile health (mHealth) to deliver an unsupervised exercise training program to a more heterogenous population of patients with all types of NAFLD [6,7]. Pfirrmann et al [6] enrolled 44 patients with NAFLD into an 8-week web-based exercise training program, in which patients completed progressive amounts of aerobic and resistance training. While exercise sessions were not directly supervised, weekly feedback was provided to individualize the exercise program and prevent injury. This web-based exercise program was feasible (74% of patients completed ≥80% of the exercise sessions) and safe (no adverse events). Modest gains in physical fitness were seen in parallel with a slight reduction in body weight (<5%) and body fat. Changes in liver fat were not reported, although transient elastography was performed, which demonstrated a small reduction in liver stiffness but no change in liver fibrosis stage.

Lim et al [7] recently explored the efficacy of Nutritionist Buddy, an mHealth app–delivered lifestyle intervention, through which patients with NAFLD were given progressive daily step goals up to 10,000 steps/day, on top of real-time dietitian support services for behavioral change. And while the authors did not provide information regarding changes in daily steps or physical activity as a whole, the mHealth intervention was successful in achieving at least 5% weight loss with corresponding improvement in metabolic parameters in 25% and 40% of patients at 3 and 6 months, respectively. However, liver-specific benefits were not measured beyond a reduction in liver enzymes; besides, change in liver fat, physical fitness, or insulin resistance were not investigated.

Another important question that remains unanswered is whether or not direct supervision by a fitness professional using telehealth outperforms unsupervised mHealth-based lifestyle modification programs. While it is plausible to imagine regular interaction with a fitness professional over any medium, including A–V telehealth technology, may lead to increased accountability and the potential for greater exercise adherence, no direct head-to-head comparison between unsupervised mHealth-based and supervised telehealth exercise training programs has been made to date. Future study investigating this question would seem of high importance given the rapidity at which telehealth is being incorporated into routine medical care and the expected increase in NAFLD and NASH after the COVID-19 pandemic resolves.

Our study has several limitations worth noting: (1) The sample size of 3 patients limits large-scale conclusions. (2) Patients were highly selected and exclusively female, mobile device literate, and English speaking which limits the generalizability of our preliminary findings. (3) While not powered to determine change in clinical outcomes, no secondary clinical outcome achieved statistical significance and future studies are required to confirm our signal of clinical efficacy.

In conclusion, our findings demonstrate proof of concept that exercise training is feasible, safe, and likely efficacious as well. These findings require validation in a larger randomized controlled trial. If validated, new telehealth-delivered exercise training programs have the possibility to increase exercise adherence and sustainability which we would anticipate to alter the natural history of NAFLD and NASH and improve patient-oriented outcomes.

## Abbreviations

A–V: audio–visual
mHealth: mobile health
MRI-PDFF: magnetic resonance imaging proton density fat fraction
NAFLD: nonalcoholic fatty liver disease
NASH: nonalcoholic steatohepatitis
